# The Immunomodulatory Functions of Various CpG Oligodeoxynucleotideson CEF Cells and H_9_N_2_ Subtype Avian Influenza Virus Vaccination

**DOI:** 10.3390/vaccines10040616

**Published:** 2022-04-14

**Authors:** Chenfei Li, Xiangyu Huang, Jiaxi Cai, Anran Lu, Shanshan Hao, Ze Zhang, Haifeng Sun, Xiuli Feng

**Affiliations:** 1 Key Laboratory of Animal Microbiology of China’s Ministry of Agriculture, College of Veterinary Medicine, Nanjing Agricultural University, Nanjing 210095, China; 2019807121@njau.edu.cn (C.L.); 2020107044@stu.njau.edu.cn (X.H.); 2018807121@njau.edu.cn (J.C.); 2019107046@stu.njau.edu.cn (A.L.); 2017107046@stu.njau.edu.cn (S.H.); 2018107042@stu.njau.edu.cn (Z.Z.); shf@njau.edu.cn (H.S.); 2 MOE Joint International Research Laboratory of Animal Health and Food Safety, College of Veterinary Medicine, Nanjing Agricultural University, Nanjing 210095, China

**Keywords:** CpG ODN, CEF, immune regulation, chicken immunization experiments, H_9_N_2_ inactivated vaccine

## Abstract

CpG oligodeoxynucleotides (CpG ODN) present adjuvant activities for antigen proteins, which can induce humoral and cellular immune responses to antigens. However, the immunomodulatory functions of CpG ODNs with different sequences are very different. In this paper, six CpG ODNs with different sequences were designed based on CpG2007 as a template. Through the screening of CEF cells in vitro, the stimulating activity of CpG ODNs was determined. Then, two selected CpG ODN sequence backbones were modified by substituting the oxygen with sulfur (S-CpG) and verifying the immune activity. Next, to prove the feasibility of S-CpG as an immune potentiator, two immune models with or without white oil adjuvant were prepared in 20-day-old chicken vaccinations. The screening experiment in vitro showed that the inducing roles of CpG ODN 4 and 5 could strongly stimulate various immune-related molecular expressions. Additionally, CpG ODN 4 and 5 with sulfation modification significantly induced various cytokines’ expressions. Furthermore, CpG ODN 4 and 5 induced the strongly humoral and cellular immune responses during vaccination, in which white oil, as an adjuvant, could significantly improve the immune effect of CpG ODN. These results provide an important experimental basis for exploring the structural characteristics and vaccine immunity of CpG ODN.

## 1. Introduction

CpG oligodeoxynucleotides(CpG ODNs) are a pathogen-related molecular model (PAMP) with a variety of immunostimulatory activities, which could induce strong humoral and cellular mediated immunity [1,2,3]. Additionally, CpG ODNs have anti-viral, anti-infection, anti-allergic, and anti-tumor effects. Because of their low toxicity and immunomodulatory function, CpG ODNs have been widely used to prevent and control human and animal-related diseases.

Currently, CpG ODNs are classified into three types: A, B, and C, based on their variability in structure and immune function [4,5]. Type-A CpG ODNs have a chimeric backbone structure, in which the core region with a palindrome structure in the middle is a phosphate skeleton, and both ends or the polyguanylate region at the 3’ end only are a thiophosphate skeleton. Type-A CpG ODNs stimulate NK cells and plasma cell-like DCs (pDCs) to secrete IFN-γ and IFN-α, respectively [6,7]. The skeleton structure of Type-B CpG ODNs was full-text this-modification, and the 5′ terminal was TCGTT or TCGTC, in which the number of CpG dinucleotides affects the stimulating activity of ODN. Moreover, type-B CpG ODNs stimulate monocytes and DCs to secrete IL-6 and promote B cell proliferation, activation, and secretion of IgM and IL-6 [4]. Backbone sulfation modification enhances the resistance of type B CpG ODN to nuclease digestion, thereby prolonging the duration of action in vivo [8]. The main features of type-C CpG ODNs include one or two TCG motifs in the 5′ end, the whole sequence with the palindrome structure containing at least two CpG dinucleotides composed of 10–12 bases, and CpG dinucleotides separated by bases from zero to three. Furthermore, type-C CpG ODNs share the biological functions of both A and B-type CpG ODNs [9]. It was proven that CpG ODNs, as an adjuvant, protect chickens from various diseases, including Newcastle disease, H_9_N_2_ and H_7_N_9_ avian influenza, salmonellosis, and colibacillosis [10,11].

CpG ODNs present species-dependent and structure-dependent features. The pattern recognition receptor recognized by CpG ODNs in mice and humans is TLR-9, and TLR-21 in poultry [12,13]. CpG2007 has been reported to tend to activate human immune cells [14,15]. However, the functions of CpG ODNs based on avian cells and animal immunization have been little reported. The main factors affecting the immunostimulatory activity of CpG ODNs might include the CpG skeleton structure, CpG flanking sequence, terminal polyG modification, and the number and location of CpG motifs [16]. Therefore, in this paper, we designed some CpG ODN sequences by changing the TCGTCGTT location and adding interval CG sequences (Table 1). Simply, the five-terminal sequence TCGTCGTT of CpG2007 was retained, and several CG-rich sequences were added. Additionally, in CpG3, TT was inserted, which enabled five-terminal and intermediate sequences to be the same, and the three-terminal TCG motif was retained. In CpG5, three-terminal sequences were replaced with GGGGGG [4,5].We first screened the inducing roles of the six designed CpG ODNs on the immune molecules of avian CEF cells. Subsequently, we compared the inducing effect of three pre-screened CpG ODNs with thio-modification on cytokines secreted by chick embryo fibroblasts (CEF) and investigated the immunomodulatory functions as an adjuvant on the H_9_N_2_ avian influenza virus (AIV) vaccine. This study provided experimental data for the structural function of CpG ODN and clinical applications.

## 2. Materials and Methods

The experiment was carried out in the Animal Disease Genetic Engineering Laboratory, School of Veterinary Medicine, Nanjing Agricultural University, Jiangsu Province from October 2019 to November 2020.

### 2.1. CpG ODNSynthesis, CEF and Chickens

The designed six CpG ODNs with different sequences and CpG2007 were listed in Table 1, and were synthesized by Sangon Biotech (Shanghai, China). Additionally, three CpG ODN sequence backbones were modified by substituting the oxygen with sulfur, namely thio-modification (S-CpG), as listed in Table 1.

CEF cells were prepared from 9-day-old SPF chicken embryos purchased from Jinan Seth Poultry Technology (Jinan, China) and were cultured in DMEM medium (Gibcol) with 10% fetal bovine serum (FBS, Gibcol).

The 20-day-old HY-LINE VARIETY BROWN chickens were purchased from Nanjing Special Power Planting Cooperative (Nanjing, China) and reared in the Experimental Animal Center of Nanjing Agricultural University (Nanjing, China).

### 2.2. Cell Treatment and MTT Assay

The CEF cells were adjusted to 6 × 10^5^ cells/mL and cultured in 5% CO_2_ at 37 °C. The cells were grown to the logarithmic growth phase and were treated with six CpG and three S-CpG at experimental dosages, respectively. Concurrently, CpG2007 was used as a positive control, PBS as the negative control, and the cell-free DMEM as the blank control.

At 48 h post-incubation, 20 μL MTT (A600799-0001,Sangon Biotech, Shanghai, China) was added to each well and incubation continued for 4 h. Then, 150 μL DMSO (A100231-0500, Sangon Biotech, Shanghai, China) was added to dissolve the crystalline violet completely. The optical density values of each well at a 630 nmwavelength (OD630) were measured using a detector (M8180, Solarbio, Beijing, China). The data of each group were analyzed by the stimulation index (SI), where SI = (OD experimental group-OD blank control)/(ODPBS group-OD blank control).

### 2.3. qPCR

At 3, 6, and 12 h after treatment with six CpGs and three S-CpGs, the total RNA was extracted by TRIzol lysis and cDNA, incubation synthesized by reverse transcription, and then fluorescence quantitative PCR (qPCR) (639676, Takara, Dalian, China) was used to detect the relative expression levels of cytokines of CEF (IL-2, IL-6, IL-12 IFN-α, and IFN-γ) and the pattern recognition receptor TLR-21. The special primer sequences were shown in Table 2. The PCR procedure was as follows: 95 °C for 60 s, 95 °C for 10 s, 60 °C for 30 s, and 35 cycles. Data of the qPCR results were calculated using the 2-ΔΔCq method [17], ΔΔΔCq = ΔCq (Cq target gene-Cq internal reference gene) in the test group-ΔCq (Cq target gene-Cq internal reference gene) in the control group, in which β-actin was the internal reference gene.

### 2.4. Inactivated Vaccine Preparation and Chicken Immunization

H_9_N_2_ AIV antigen was harvested from the allantoic fluid infected with the virus for 20 h and was inactivated with formaldehyde at a final concentration of 1‰ for 24 h. Inactivated virus antigens and S-CpG ODN 4 or 5 were completely mixed and emulsified with white oil adjuvant(Montanide ISA 206 VG) based on the volume ratio of 1:1. Additionally, PBS and CpG2007 were used as the control. The immunization procedure was listed in Table 3.

The 100 20-day-old HY-LINE VARIETY BROWN chickens were randomly divided into 10 groups. The chickens were subcutaneously immunized with 0.25 mL of the prepared inactivated vaccine, followed by two booster immunizations at an interval of a fortnight. 14 days after each immunization, serum samples were collected from the immunized chickens to detect the antibody level.

### 2.5. HI Antibody Tests

HI experiments were used to detect the antibody level following experimental guidance. Simply, the serum to be tested was diluted multiplicatively, and an equal volume of four units of H_9_N_2_ virus antigen was added. After mixing thoroughly and storing at room temperature for 30 min, an equal volume of 1% chicken red blood cells was added to the wells for 30 min. The result determination was as follows. The highest dilution of the serum that completely inhibited the agglutination of red blood cells was used as the HI titer. HI antibody titers with less than and including 3log2 were considered below the protection level, and more than and including 4log2 were considered to reach the protection level.

### 2.6. MTT Assay

The splenic lymphocytes were isolated from the immunized chickens and were added to 96-well cell plates in a 5% CO_2_ at 37 °C. When they grew to the logarithmic growth phase, LPS and PBS were added to the splenic lymphocytes for stimulation. After 48 h, MTT was added to the cells for 6 h, and the cells were then lysed with DMSO. Then, data were analyzed to detect the spleen lymphocyte proliferation viability.

### 2.7. ELISA Assays

The cell supernatants of splenic lymphocytes cultured for 48 h were collected, and the expression levels of IL-4 (MM-0527O2, Meimian, Nanjing, China) and IFN-γ (MM-0520O2, Meimian, Nanjing, China), and the levels of IgM (MM-0912O1, Jiangsu Meimian, China) antibodies secreted by lymphocytes were detected according to the instructions of the ELISA kit according to the guidance.

### 2.8. Molecular Assay for Spleen Lymphocyte Surface Activity

Spleen lymphocytes were isolated from the immunized chickens, and the total RNA was extracted following the TRIzol instructions. The relative expressions of active molecules CD3, CD4, CD8, CD80, CD86, CD154, and BAAF in spleen lymphocytes were detected by qPCR(639676, Takara, Dalian, China), and the primers of these genes are listed in Table 4.

### 2.9. Statistics Analysis

The data were analyzed by one-way ANOVA using GraphPad Prism program (version 6.01, Graph Pad Software Inc., La Jolla, CA, USA), and Duncan’s post hoc test was used for multiple comparisons. The results were expressed as the mean ± standard deviation. *p*-value less than 0.05 with an asterisk indicated a significant difference.

## 3. Results

### 3.1. Viabilities of CpG on CEF

**Figure 1:**
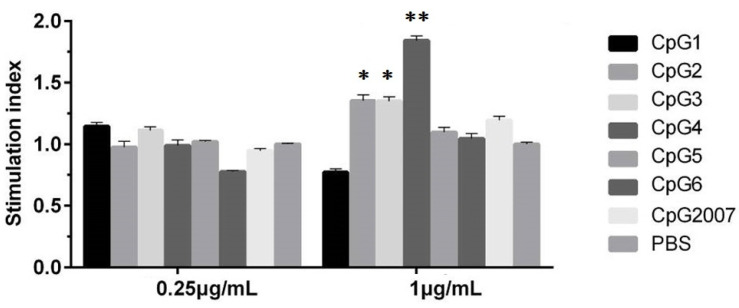
Proliferation viabilities of CpG on CEF. CEF were treated with 0.25 and 1 μg/mL six designed CpGs and CpG2007, and viabilities were detected by the MTT assay. All data were presented as mean ± SD. * *p* < 0.05, ** *p* < 0.01.

Firstly, the cell proliferation effect of CpG at experimental concentrations on CEF was examined by the MTT assay. As shown in Figure 1, the CEF viabilities treated with 1 μg/mL of CpG ODN 2, 3, and 4 groups were higher than those of the PBS control, with the highest viability in the CpG ODN 4 group (*p* < 0.01), which was also higher than that of CpG2007. However, compared with that of the PBS control, there was no significant effect of the six CpG ODNs and CpG2007 on cell viability at 0.25 μg/mL. These results indicate that CpG ODN at both experimental concentrations had no cytotoxic effect on CEF.

### 3.2. CpG Induced Cytokines Expression in CEF after Treatment

**Figure 2:**
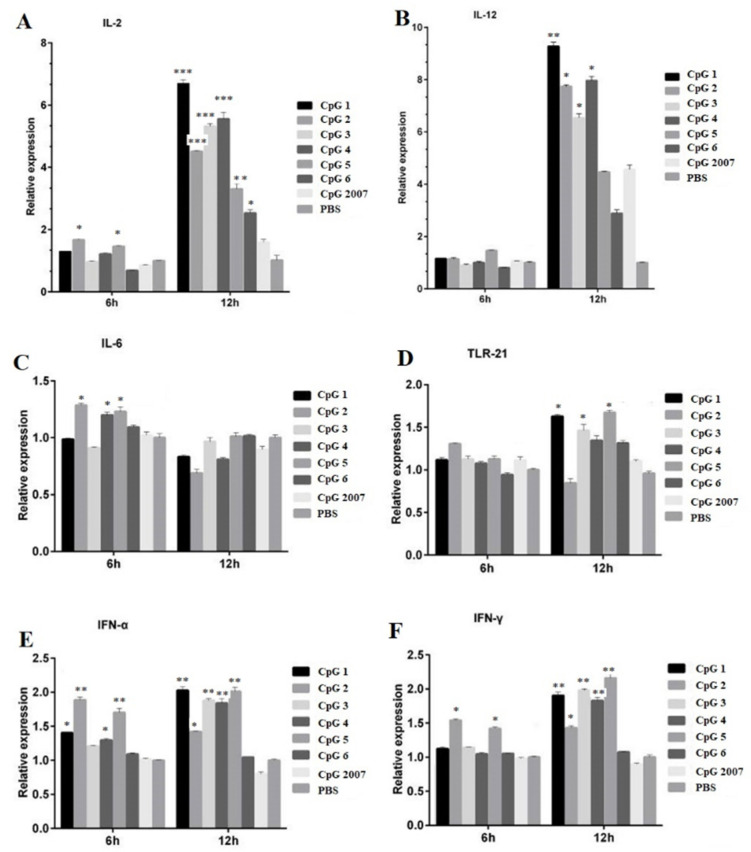
Effect of CpG on the mRNA expression of CEF related immune genes. CEFs were treated with 0.25 μg/mL six CpGs and CpG2007 for 6 and 12 h. The expressions of IL-2 (**A**), IL-12 (**B**), IL-6 (**C**), TLR-21 (**D**), IFN-α (**E**) and IFN-γ (**F**) were detected with qPCR. All data were presented as mean ± SD. * *p* < 0.05, ** *p* < 0.01, and *** *p* < 0.001.

To investigate the roles of six designed CpG ODNs on immune-related active molecules, in the paper, five cytokines and TLR-21 expressions in CEF treated with 0.25 μg/mL CpG ODNs were detected by qPCR, respectively. As shown in Figure 2A,B, compared with that of the CpG2007 control, the IL-2 expressions in the CEF with 0.25 μg/mL CpG ODN 1, 2, 3, 4, 5, and 6 treatments were significantly increased at 12 h, and the IL-12 expressions in the CEF with 0.25 μg/mL CpG ODN 1, 2, 3 and 4 treatments were significantly increased at 12 h. The IL-2 expressions with 0.25 μg/mL CpG ODN 2 and 5 at 6 h after treatment were significantly higher than that of the CpG2007 control.

The IL-6 expression in the CEF treated with 0.25 μg/mL CpG 2, 4, and 5 was higher than that of the CpG2007 control (Figure 2C). Additionally, the TLR21 expressions treated with 0.25 μg/mL CpG 1, 3,and 5 at 12 h after treatment were significantly higher than that of the CpG2007 control (Figure 2D). As for alpha IFN, at 6 h, the expression with 0.25 μg/mL CpG 1, 2, 4, and 5 was significantly higher than that of the CpG2007 control, and at 12 h, the expressions with 0.25 μg/mL CpG 1, 2, 3, 4, and 5 were higher than that of the CpG2007 control (Figure 2E). Meanwhile, at 6 h, the levels of IFN-γ in CpG ODN 2 and 5 were significantly higher than that of the CpG2007 control, while, at 12 h, the levels of IFN-γ in the CpG ODN 1, 2, 3, 4, and 5 groups were significantly higher than those in the CpG2007 control group (Figure 2F).

These results suggested that the six designed CpG ODNs might possess different regulatory functions for immune-related active molecules in CEF. Based on these results, CpG ODN 4 and 5 were screened from the six designed CpG ODNs to further investigate the immunomodulatory functions.

### 3.3. Viabilities of S-CpG on CEF

**Figure 3:**
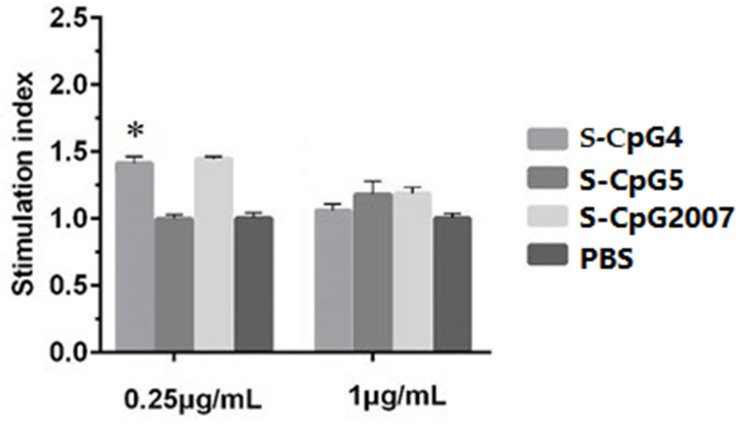
Cell proliferation test of CEF by S-CpG. CEF were treated with 0.25 and 1 μg/mL S-CpG4, 5 and S-CpG2007, and viabilities were detected by the MTT assay. All data were presented as mean ± SD. * *p* < 0.05.

Thio-modification has been reported to be a common modification of CpG ODN [18]. To verify the immunostimulatory effect of CpG ODN with thio-modification, in this paper, the pre-selected CpG ODN 4, 5, and CpG2007 were subjected to thio-modification. As shown in Figure 3, the results showed that the cell viability of 0.25 μg/mL S-CpG ODN 4 was significantly higher than that of the PBS control, which was similar to that of CpG2007, while the cell viability of the S-CpG ODN5 group was similar to that of the PBS control. When the dose was 1 μg/mL, the cell viabilities of the three S-CpG ODNs were similar to that of the PBS control, with no significant difference. These results indicated that S-CpG ODN at the concentration gradient of this experiment might have no cytotoxic effect on CEF.

### 3.4. S-CpG Stimulated the Expressions of Immune-Related Genes in CEF

**Figure 4:**
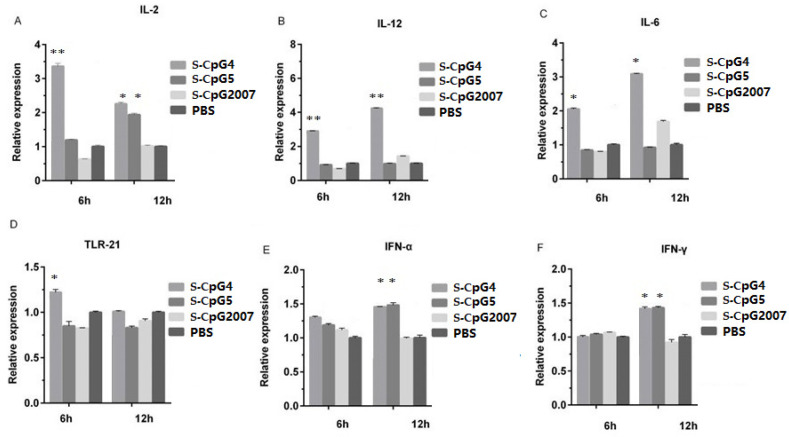
Effect of S-CpG on the mRNA expression of CEF-related immune genes. CEFs were treated with 1 μg/mL S-CpG4, 5 and S-CpG2007 for 6 and 12 h. The expressions of IL-2 (**A**), IL-12 (**B**), IL-6 (**C**), TLR-21 (**D**), IFN-α (**E**) and IFN-γ (**F**) were detected with qPCR. All data were presented as mean ± SD. * *p* < 0.05, ** *p* < 0.01.

To further verify the regulatory function of the three S-CpG ODNs on immune-related molecules, 1 μg/mL was used to stimulate the CEF. The levels of IL-12, IL-6, and TLR-21 in the CEF treated with S-CpG ODN 4 were significantly higher than that of the CpG2007 control at 6 h (Figure 4B–D: 6 h), and the levels of IL-2 and IFN-α in the CEF treated with the S-CpG ODN 4 group were significantly higher than that of the CpG2007 control (Figure 4A,E: 4 h). When the CEF was stimulated for 12 h, the intracellular levels of IL-2, IFN-α, and IFN-γ in the CEF treated with S-CpG ODN4 and 5 were significantly higher than that of the CpG2007 control (Figure 4A,E,F: 12 h), and the intracellular levels of IL-12 and IL-6 were significantly higher in the S-CpG ODN 5 groups than the CpG2007 control (Figure 4B,D: 12 h). These results suggest that the sulfur-modified CpG ODN 4 and 5 have strong abilities to regulate the active molecules in CEF cells at 1 μg/mL.

### 3.5. CpG ODN Stimulated HI Antibody Production

**Figure 5:**
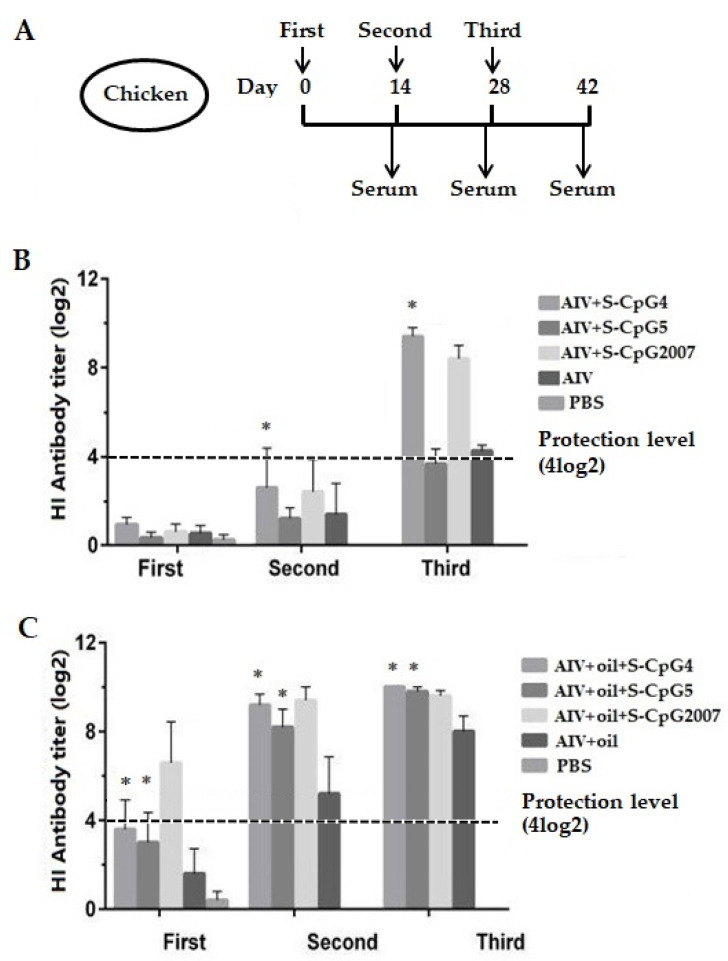
Antibody levels of the immunized chickens. (**A**) Scheme of immunization (n = 10/group) and samples. (**B**,**C**) Antigen-specific serum antibody titers of chickens with S-CpGs and AIV (**B**), and with S-CpGs and AIV and oil adjuvant (**C**) were measured with HI method. All data were presented as mean ± SD. * *p* < 0.05.

To study the immune adjuvant effects of CpG ODNs on animal vaccination in vivo, two S-CpG ODNs were mixed with inactivated AIV antigen or with white oil adjuvant to be injected into 20-day-old chickens with three immunizations, and the serum was collected three times at an interval of a fortnight (Figure 5A) to detect the antibody levels.

As shown in Figure 5B, all immunized chickens without the addition of white oil adjuvant produced very low HI antibody levels at the first immunizations. At the second and third immunization, the levels of HI antibodies produced in the chickens immunized with AIV antigen and S-CpG ODN 4 were significantly higher than that of the AIV antigen control, and were higher than of CpG2007 at the third immunization.

As for the oil adjuvant model, at two weeks after the first and second vaccinations, the levels of HI antibodies produced in chickens immunized with two S-CpG ODN experimental groups were significantly higher than that of antigen control, which was lower than that of CpG2007 (Figure 5C, first and second). Furthermore, the HI antibodies produced in the chickens of the two S-CpG ODN experimental groups of the third vaccination were similar to those of CpG2007 (Figure 5C, third).

In this paper, 4log2 was taken as the critical boundary of immune protection in the antibody level of immunized chickens [19]. Additionally, it was observed that the antibody levels of S-CpG ODN, as an immune potentiator, increased with the increased immunization times, in which the antibody levels of chickens immunized by S-CpG with or without oil adjuvant were increased after the third immunization (Figure 5).

### 3.6. CpG ODN Induced Cytokine and Specific Antibody Production

**Figure 6:**
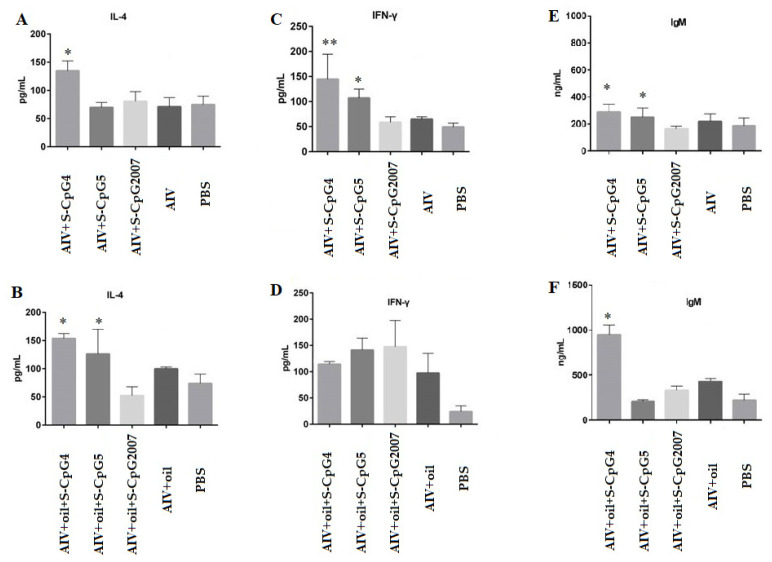
Expression levels of IL-4, IFN-γ, and IgM secreted by splenic lymphocytes. The expressions of IL-2 (**A**), IFN-γ (**C**) and IgM (**E**) of splenic lymphocytes from immunized with S-CpG4, 5 and S-CpG2007 and AIV antigen were detected with qPCR, and expressions of IL-2 (**B**), IFN-γ (**D**) and IgM (**F**) from immunized with S-CpG4, 5 and S-CpG2007 and AIV antigen with oil adjuvantt were measured with qPCR. All data were presented as mean ± SD. * *p* < 0.05, ** *p* < 0.01.

To investigate the function of CpG ODN in the cytokine and specific antibody responses, in this paper, spleen lymphocytes were isolated to detect the production of cytokines IL-4 and IFN-γ, as well as IgM antibodies. The results showed that the IL-4 level of chickens immunized with AIV antigen and S-CpG ODN 4 was 134.75 pg/mL, which was significantly higher than that of theCpG2007 control (Figure 6A). Additionally, the production of IL-4 from AIV antigen plus white oil combined with S-CpG ODN 4 and 5 was higher than that of CpG2007 plus white oil immunization control (Figure 6B), in which the S-CpG ODN4 immunization group produced the highest IL-4 levels among all experimental groups. Additionally, the elevations of the IFN-γ levels from the CpG ODN 4 and 5 experimental groups were higher than that of the CpG2007 control (Figure 6C). However, the production of IFN-γ from S-CpG 4 was similar to that of the CpG2007 plus oil control (Figure 6D).

Furthermore, it was observed that the production of IgM from AIV antigen with two S-CpG was significantly higher than that of the CpG2007 control (Figure 6E), and the AIV antigen plus white oil combined with S-CpG ODN 4 experimental groups produced 949.59 ng/mL of IgM, which was significantly higher than that of the CpG2007 plus oil control (431.07 pg/mL, Figure 6F).

These data suggest that the thio-modified CpG ODN 4 and 5 might be able to stimulate the cytokine and antibody responses in immunized chickens, in which white oil adjuvant might play an important role in the inducing function of CpG ODNs, based on the antigen model.

### 3.7. CpG ODN Stimulated theT Lymphocyte Molecular Expressions

**Figure 7:**
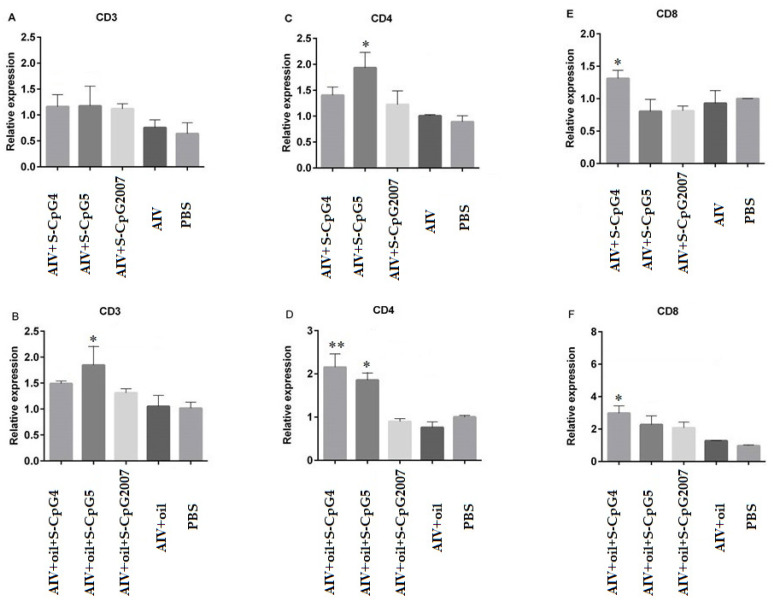
Expression levels of CD3, CD4, and CD8 on splenic lymphocytes. The expressions of CD3 (**A**), CD4 (**C)** and CD8 (**E**) of splenic lymphocytes from immunized with S-CpG4, 5 and S-CpG2007 and AIV antigen were detected with qPCR, and expressions of CD3 (**B**), CD4 (**D**) and CD8 (**F**) from immunized with S-CpG4, 5 and S-CpG2007 and AIV antigen with oil adjuvantt were measured with qPCR. All data were presented as mean ± SD. * *p* < 0.05, ** *p* < 0.01.

To detect the roles of CpG ODNs in cellular mediated immune responses, in this paper, the molecules CD3, CD4, and CD8 in lymphocytes isolated from immunized chickens were detected by qPCR. The results showed that, although the expressions of CD3 molecules from two S-CpG ODN and AIV antigen groups were similar to that of the CpG2007 control (Figure 7A), the expressions of CD3 molecules from S-CpG ODN5 were significantly higher than that of the CpG2007 plus oil control (Figure 7B). Additionally, the expressions of CD4 molecules from the AIV antigen and S-CpG ODN 5 groups were significantly higher than that of the CpG2007 control (Figure 7C). Compared to that of the CpG2007 plus oil control, the expressions of CD4 molecules from the S-CpG ODN 4 and 5 groups were significantly increased (Figure 7D), in which the CD4 molecule expressions of S-CpG ODN 4 were highest. Additionally, the expressions of CD8 molecules from the S-CpG ODN 4 groups and AIV antigen with or without white oil and were significantly higher than that of the CpG2007 control (Figure 7E,F), in which S-CpG ODN 4 produced the highest expressions of CD8 molecular among all of the immunized chickens.

### 3.8. CpG ODN Stimulated Antigen Presentation Related Molecular Expressions

**Figure 8:**
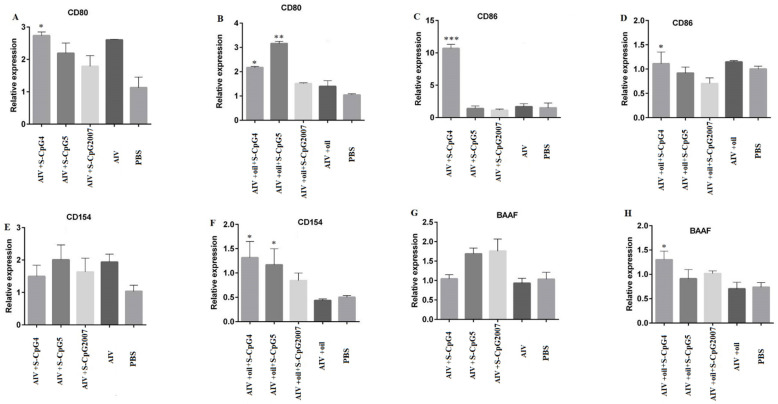
Expression levels of CD80, CD86, CD154, and BAAF on splenic lymphocytes. The expressions of CD80 (**A**), CD86 (**C**), CD154 (**E**) and BAAF (**G**) of splenic lymphocytes from immunized with S-CpG4, 5 and S-CpG2007 and AIV antigen were detected with qPCR, and expressions of CD80 (**B**), CD86 (**D**), CD154 (**F**) and BAAF (**H**) from immunized with S-CpG4, 5 and S-CpG2007 and AIV antigen with oil adjuvant were measured with qPCR. All data were presented as mean ± SD. * *p* < 0.05, ** *p* < 0.01, and *** *p* < 0.001.

To investigate the role of CpG ODN in antigen presentation during vaccination, in this paper, the expressions of CD80, CD86, CD154, and BAAF in the splenic lymphocytes from the immunized chicken were detected by qPCR. The CD80 and CD86 expressions of S-CpG ODN 4 and antigen were significantly higher than that of the CpG2007control(Figure 8A,C). Additionally, it was observed that the expressions of CD80 and CD154 in the splenic lymphocytes from the chicken immunized with antigen plus oil and S-CpG ODN 4 or 5 were significantly increased compared to that of CpG2007 and antigen plus oil control (Figure 8B,F).Additionally, chickens immunized with S-CpG ODN 4 and antigen plus white oil induced significant CD86 and BAAF expression, compared to that of the CpG2007 control (Figure 8D,H).

## 4. Discussion

Both bacterial DNA and synthetic CpG ODNs stimulate vertebrate-specific and non-specific immune systems and provide immune protection against invasion by pathogenic microorganisms [20,21,22]. It has been reported that CEF is highly tolerant to the cytotoxicity of nucleic acid sequences, responsive to changes in nucleic acid structure, and easy to culture, which makes CEF very suitable for the screening of CpG ODN in vitro [23]. In this paper, we designed six different CpG ODN sequences to explore the effect of different nucleic acid sequence alterations on the immune regulatory function.

It was found that six CpG ODNs did not induce significant viabilities at 0.25 μg/mL, while CpG ODN 2, 3, and 4 induced increased viabilities at 1 μg/mL. To avoid the differences in the immune molecules caused by the difference in the CEF number, 0.25 µg/mL was used as the experimental dose for screening CpG in vitro. The screening experiment in vitro showed that the inducing roles of CpG ODN 4 and 5 could strongly stimulate various immune-related molecular expressions. Type-A CpG ODN could stimulate NK cells and plasma cell-like DCs to secrete IFN-γ and IFN-α, respectively, whereas type-B CpG ODN stimulated monocytes and DCs to secrete IL-6 and promoted B cell proliferation activation and IgM and IL-6 secretion [6,9]. In this study, CpG ODN4 belonged to B-type ODNs, and CpG ODN5 had the polyguanine nucleotide (poly Gs) structure of A-type ODNs. It has been reported that, since the poly Gs structure interacts with scavenger receptors, ODNs with this structure increase the concentration entering oligonucleotides through receptor-mediated endocytosis and deliver them to the nuclear endosomes to bind to Toll-like receptors, thereby promoting the expression of relevant immune molecules [24,25,26,27]. These results suggest that CpG ODN 4 and 5 might present an inducing function on immune activation.

Backbone sulfation modification enhanced the resistance of ODN to nuclease digestion, thus prolonging the duration of in vivo action [28]. It was found that the viabilities of CpG ODN 4 with or without thio-modification were different at experimental dosages. To avoid the differences in the immune molecules caused by the difference in the CEF number, the concentration of 1 μg/mL which did not affect the cell viability as the experimental dose was used to study the inducing roles of CpG ODN 4 and 5 with sulfation modification. It was proven that S-CpG ODN 4 significantly induced the IL-2, IL-12, IL-6, TLR-21, alpha, and gamma IFN expressions, while S-CpG ODN 5 only stimulated the IL-2, alpha, and gamma IFN expressions. Although there was no significant influence on the inducing role of CpG ODN 4 at 1 μg/mL, we speculated that sulfation modification might influence the induction of CpG ODN, in which the potential mechanism needs to be further explored.

To further verify the immunomodulatory effect of CpG ODN, two immunization models were designed, namely, inactivated H_9_N_2_ virus antigen and antigen with white oil as a supplement to enhance the immune effect of the vaccine. The HI results showed that, in the immune model without white oil adjuvant, the HI antibody titer in the S-CpG ODN 4 experimental group reached about 9 log2 after three immunizations, which was significantly higher than that of the antigen control, and was similar to that of the S-CpG2007 control. However, in the immune model with white oil adjuvant, the level of HI antibody in chickens of S-CpG ODN 4 and 5 increased significantly two weeks after the first immunization, and the titer of HI antibody in chickens of each experimental group remained above 8 log2 after the second immunization, which was similar to that of S-CpG2007 control. It was reported that chicks immunized with CpG ODN2007 and inactivated H_9_N_2_ virus produced HI antibody titers above 8 log2 two weeks after the second vaccination [26]. These results observed in the experimental group suggested that S-CpG ODN 4 and 5 might induce a similar HI antibody response to S-CpG2007, in which white oil adjuvant might play the coordinating role.

Additionally, it was found that DNA fragments containing CpG could enhance the NK cell activity and induce IFN-γ production in mouse splenocytes in vitro [29,30,31], CpG ODN-activated NK cells produced more than 90% of the total IFN-γ produced by the early cells, and most of the later IFN-γ was provided by CD4^+^ T cells [32,33]. In this paper, compared to the S-CpG2007group, in the immune model without white oil adjuvant, S-CpG ODN 4 strongly induced IL-4 and gamma IFN production, and S-CpG ODN 5 only stimulated gamma IFN production. However, in the immune model with white oil adjuvant, both S-CpG ODN 4 and 5 induced IL-4 production, which was higher than that of S-CpG2007. Furthermore, in both immune models, S-CpG ODN 4 could induce the expression of CD8 molecules and S-CpG ODN 5 could induce the expression of CD4 in splenic lymphocytes. These results suggest that, as an immune enhancer, S-CpG ODN could activate T cells, and activated T cells subsequently secrete large amounts of IFN-γ and enhance IL-4 cytokines production, in which the inducing functions might be related to different sequences of CpG ODN, and the latent mechanism might be further studied.

Furthermore, in the immune model without white oil adjuvant, S-CpG ODN 4 and 5 strongly induced IgM production, while, in the immune model with white oil adjuvant, only S-CpG ODN 4 strongly induced IgM production. It was reported that CpG ODN could directly stimulate the activation and proliferation of B cells and produce IgM antibodies without the help of T cells [33]. These results suggest that the inducing roles of S-CpG ODN 4 on B cell activation and proliferation might be more obvious than that of S-CpG ODN 5.

Additionally, CpG ODN upregulated the expression of B cell surface co-stimulatory molecules (CD40, CD86, MHC-I, MHC-II-like molecules, etc.), thereby increasing the antigen-presenting effect of B cells [32]. In the immune model without white oil adjuvant, only S-CpG ODN 4 could induce the expressions of CD80 and CD86 molecules, while, in the immune model with white oil adjuvant, S-CpG ODN 4 and 5 promoted the CD80, CD154, and BAAF expressions. These results suggest that CpG ODN could enhance both humoral and cellular immunity.

Notably, in the immune model with white oil adjuvant, white oil as an adjuvant produced significantly higher levels of HI antibodies and surface co-stimulatory molecules CD80, CD154, and BAAF expressions. These results suggested that white oil, as an adjuvant, could significantly improve the immunostimulatory effect of CpG ODN in vaccination, which provided some constructive views on the development of vaccine immune enhancers and the improvement of immune strategies.

## 5. Conclusions

The structural alterations of the CpG ODN nucleic acid sequences were very important for its immunomodulatory function. In this paper, it was proved the structural alterations of CpG ODN by changing individual bases could affect the inducing roles of CpG ODN in the immune-related molecular in vitro screening experiments. CpG ODN 4 and 5 were proven to strongly stimulate various immune-related molecular expressions. Furthermore, two immune models showed that CpG ODN 4 and 5 could significantly enhance the humoral and cellular immune responses, in which white oil as an adjuvant could significantly improve the immune effect of CpG ODN. These results provide a basis for structural and functional studies of CpG ODN and laid the fine foundation for the clinical application of the avian influenza virus vaccine.

## Figures and Tables

**Table 1:** Sequences of CpG ODNs.

Name	Sequence(5′-3′)	Thio-Modification
CpG1	TCGGCGCGCGCCGTCGTCGTTT	
CpG2	TCGTCGTTTTCGAGGCCGTCG	
CpG3	TCGTCGTTTTGTCGTTTTGTCGT	
CpG4	TCGTCGTTTTCGTTGCGCGCCG	Full-text thio
CpG5	TCGTCGTTTTGTGTTGGGGGG	Partial thio (terminal G region)
CpG6	TCGTCGTTTTCGGCGGCCGCCG	
CpG2007(Positive control)	TCGTCGTTGTCGTTTTGTCGTT	Full-text thio

**Table 2:** Fluorescent quantitative PCR primer sequences.

Gene Name	Primer Name	Primer Sequence (5′-3′)	Primer Size (More)	Tm (°C)	Product Size (bp)	GenBank Number
IL-2	IL-2-F	ACACCGGAAGTGAATGCAAG	20	61.1	81	AY510091.1
	IL-2-R	CAAAGTTGGTCAGTTCATGGAGA	23	61.43		
IL-6	IL-6-F	AAATCCCTCCTCGCCAATCT	20	62.18	106	HM179640.1
	IL-6-R	CCCTCACGGTCTTCTCCATAAA	22	62.55		
IL-12	IL-12-F	ACTTTCCTTTGCTGCCCTTCTGG	23	60.35	90	DQ202328.3
	IL-12-R	GCTGGTGTCTCATCGTTCCACTC	23	60.47		
TLR21	TLR21-F	TCTCACAGGCGGAGGTCTTCAC	22	61.74	116	JQ042914.1
	TLR21-R	GCGAGGTTGGATGTCAGAGATGTC	24	60.06		
IFN-α	IFN-α-F	CACGACATCCTTCAGCACCTCTTC	24	60.21	87	GU119896.1
	IFN-α-R	GAGGAGGCTTTGGCGTTGGC	20	62.95		
IFN-γ	IFN-γ-F	CGACATCCTTCAGCACCTCTTCAC	24	60.21	85	FJ977575.1
	IFN-γ-R	GAGGAGGCTTTGGCGTTGGC	20	62.95		
β-actin	β-actin-F	CAACACAGTGCTGTCTGGTGG	21	61.68	205	NM_205518.1
	β-actin-R	CAAAGTTGGTCAGTTCATGGAGA	23	61.43		

**Table 3:** The formulation of the influenza vaccines.

Groups	White Oil (mL)	AIV (mL)	CpG ODN (μg)	PBS(mL)
AIV + white oil + S-CpG 4	1.3	0.9	20	0
AIV + white oil + S-CpG 5	1.3	0.9	20	0
AIV + white oil + S-CpG2007	1.3	0.9	20	0
AIV + white oil	1.3	0.9	0	0.4
PBS + white oil	1.3	0	0	1.3
AIV + S-CpG 4	0	0.9	20	1.3
AIV + S-CpG 5	0	0.9	20	1.3
AIV+ S-CpG2007	0	0.9	20	1.3
AIV +PBS	0	0.9	0	1.7
PBS	0	0	0	2.6

Note: The volumes of 20 μg CpG or S-CpG were 0.4 mL.

**Table 4:** Primers used to detect the molecules in chicken spleen lymphocyte.

Gene Name	Primer Name	Primer Sequence (5′-3′)	Primer Size (More)	Tm (°C)	Product Size (bp)	GenBank Number
CD3	CD3-F	GGACGCTCCCACCATATCAG	20	60	113	NM_205512.1
	CD3-R	AAGCTCGTGACATGAGTCCC	20	57.1		
CD4	CD4-F	TGTGGAACTGTCACCTCGTG	20	56.8	145	NM_204649.1
	CD4-R	CACATGCATGCAAGGCCAAT	20	62.4		
CD8	CD8-F	GCTGTACTTCAGCTCGGGAC	20	57.2	105	NM_205235.1
	CD8-R	ATGTCCTTGTTGACGTGGCT	20	57.5		
CD80	CD80-F	TGTGACCCTCTTTGTCACCG	20	58.7	140	NM_001079739.1
	CD80-R	GGAATCCACGGATTTCGGGT	20	63.2		
CD86	CD86-F	ACCAGCAAGCTGAATATCCCA	21	59.7	105	NM_001037839.1
	CD86-R	GACTAGCGGCACTGAGACAA	20	56		
CD154	CD154-F	TGCAGAAATGTCAGACGGGA	20	59.5	113	NM_204733.1
	CD154-R	CAACTCCTCACTGGCTGTCC	20	57.5		
BAAF	BAAF-F	CCTGCTTGCAACTGATTGCT	20	58.8	106	NM_204327.2
	BAAF-R	TCTTCCAGAGCTGTTCCACG	20	58.4		
β-actin	β-actin-F	GAGAAATTGT GCGTGACATCA	21	56.6	152	NM_205518.1
	β-actin-R	CCTGAACCTCTCATTGCCA	19	58.3		

## Data Availability

The data presented in this study are available upon request from the corresponding author.
